# The Effect of Thermocycling on Interfacial Bonding Stability of Self-Etch Adhesives: OCT Study

**DOI:** 10.1155/2021/5578539

**Published:** 2021-06-08

**Authors:** T. A. Bakhsh, A. Turkistani

**Affiliations:** ^1^Department of Restorative Dentistry, Faculty of Dentistry, King Abdulaziz University, P.O. Box 80209, Jeddah 215-89, Saudi Arabia; ^2^Cariology and Operative Dentistry Department, Tokyo Medical and Dental University, 1-5-45 Yushima, Bunkyo-ku, Tokyo 113-8549, Japan

## Abstract

**Objective:**

The aim of this study was to monitor the behavior of interfacial gaps formed under different bonded polymeric restorations before and after thermocycling (TC), using swept-source optical coherence tomography (SS-OCT) and confirming the obtained findings with confocal laser scanning microscopy (CLSM).

**Materials and Methods:**

Cylindrical class I cavities were prepared in twenty noncarious human premolar teeth (1.5 mm depth × 3.5 mm diameter) and divided randomly into two groups: TS and SN, according to the adhesive system (*n* = 10). In the TS group, one-step self-etch adhesive Clearfil Tri-S Bond Plus (Kuraray Noritake Dental, Japan) was used, followed by composite restoration using Estelite Sigma Quick (Tokuyama Dental, Japan). In the SN group, the cavities were restored with the two-step self-etch/composite silorane-based resin restoration system (3M ESPE, USA). All specimens were restored in bulk filling technique and cured in accordance with the manufacturers' instructions. Both groups were imaged under SS-OCT after 24 h and recorded as controls. Then, each group was subjected to thermal challenge using the TC machine (5–55°C) and B-scans were recorded at different TC intervals (2600, 5200, and 10000). In order to confirm the SS-OCT findings, additional specimens were prepared, scanned, and sectioned for CLSM observation.

**Results:**

B-scans demonstrated white clusters at the tooth-resin interface that corresponded to the gap location on CLSM images. The TS group showed significantly less gap formation than the SN group before and after TC (*p* < 0.001).

**Conclusions:**

An optimal composite adaptation can be achieved when the bonded restoration comprises a combination of an adhesive containing 10-MDP monomer and a considerable highly filled composite.

## 1. Introduction

Adhesive dentistry has been continuously developed, attempting to simplify the restorative procedure and reducing resin polymerization shrinkage as well. Many of the newly introduced materials showed satisfactory immediate sealing results. However, the durability of sealed interfaces over time is still a challenge. In clinical situations, the restoration is subjected to mechanical and thermal stresses of the oral cavity, challenging the long-term durability of the adhesive layer and the composite adaptation [1–3]. Bond durability is critical for the long-term success of composite restoration, especially in restorations with dentin margins [4–6]. To anticipate the long-term performance of newly introduced materials, aging by simulating stressful oral conditions in vitro or water storage has been used [7, 8]. One of the main methods utilized in adhesive tests is thermal cycling or thermocycling (TC) [9]. Dental restorations in the oral cavity are subjected to TC because of the intermittent temperature changes as a result of the day–night cycles [8]. Usually, the adhesive layer subjected to TC undergoes cycles of high and low stresses at different temperatures [10]. This is attributed to the apparent differences in the thermal expansion coefficient of adhesive and bonded substrates. Consequently, damage and debonding may occur due to the residual stresses in the polymeric layer, which are generated over time as a result of the viscoelastic response. This would reduce bond durability and occasionally lead to restoration failure [10].

Optical coherence tomography (OCT) is a well-established imaging system in biomedical fields [11]. In dentistry, OCT has been an attractive tool for monitoring and long-term studies [12–14]. The reliance on backscattered light from tissue structure to produce real-time images has allowed high-resolution subsurface evaluation without destructing the specimen. OCT has been successfully used to monitor dental restoration and the progression of demineralization or remineralization of lesions [15–20].

The objective of the study was to monitor the behavior of interfacial gaps formed under different bonded polymeric restorations before and after TC using swept-source OCT (SS-OCT) and confirm the obtained findings with confocal laser scanning microscopy (CLSM). The null hypothesis was that the percentages of interfacial gaps in bonded restorations with one-step and two-step self-etch adhesives do not change after TC.

## 2. Materials and Methods

### 2.1. Tested Materials

The two adhesive systems used in this study were one-step self-etch adhesive Clearfil Tri-S Bond Plus (TP; Kuraray Noritake, Japan) and two-step self-etch silorane adhesive system (SA; 3M ESPE, USA). Two dental composites were used in this study: Estelite Sigma Quick (EQ; Tokuyama Dental, Japan) and Filtek Silorane composite (FS; 3M ESPE, USA). The chemical compositions of the used materials and method of application are listed in Table 1.

### 2.2. Specimen Preparation

The study was approved by the Research Ethics Committee of King Abdulaziz University (code: 068-16), which is in accordance with the guiding principles for investigational methods found in the Declaration of Helsinki of the World Medical Association. Based on a pilot study, sample size calculation was made using a 0.05 alpha value and 80% power to detect a difference of 25% (PiFace, http://homepage.stat.uiowa.edu/~rlenth/Power/). The common standard deviation within a group was assumed to be 18%. The estimated sample size for each group should be at least 9 [21]. As a result, twenty extracted noncarious human premolar teeth were finally selected and preserved for 2 weeks in distilled water at 4°C before beginning the experiment. The roots were removed at the cementoenamel junction, and the remaining crown was cleansed from tissue debris and calculus. After a slight reduction of the cusps, cylindrical class I cavities were prepared on the occlusal surface (1.5 mm depth × 3.5 mm diameter) and the cavity floor being located in dentin. Each specimen was placed in a vial and given a code. Then, they were distributed equally into two groups: TS and SN. This was carried out using simple random sampling technique, which involves equal and random distribution of the specimens into two experimental groups.

In the TS group, the prepared cavities were bonded with the TP adhesive, photoactivated for 10 s, and restored with the EQ composite that was light cured for 10 s. The SA adhesive and the FS composite were used to restore the cavities of the SN group. The light curing time of the SA adhesive was 10 s while it is 20 s for the FS composite restoration. All materials were applied and photoactivated according to the manufacturers' recommendations (Table 1) using a halogen curing unit (Optilux501, Kerr, USA; 500 mW/cm^2^ intensity).

In order to maintain the reproducibility of the restoration process, material application and photoactivation were carried out according to the previously described protocol in prior studies [15, 22, 23]. After preparing the cavities, each specimen was held vertically to avoid pooling the adhesive to one side of the cavity during the bonding procedure. Before dispensing the adhesive bond into the mixing well, the adhesive bottle was shaken to ensure complete binding of the adhesive components. Then, using a disposable microbrush, the adhesive was applied in a rubbing motion. The air-drying step was performed while a 3-way syringe was held vertically over the bonded cavity at a 20 cm distance to remove the excess adhesive, obtain a uniform adhesive layer, and ensure the solvent's evaporation [24]. Photoactivation of the applied adhesive was accomplished while the light guide tip of the light cure was positioned within a 2 mm distance from the occlusal surface and continuous irradiation was performed. Next, a small volume of the composite was squeezed out of the composite tube and covered by a plastic dappen dish to protect the composite from premature polymerization. Afterward, a plastic filling instrument was used to fill the prepared cavity with one increment of dental composite in bulk filling technique. After compacting the composite and removing the excess, each specimen's occlusal surface was covered with a glass slide to standardize the packing pressure and improve composite structural integrity while being photopolymerized. Composite photoactivation was conducted similar to the above-mentioned adhesive light-curing protocol. The light-guide tip of the light cure was positioned within 2 mm distance from the restoration surface, and continuous irradiation was performed. These aforementioned steps were strictly followed to standardize the restorative procedure in both groups and ensure the adequacy of the assessed outcome.

**Figure 1:**
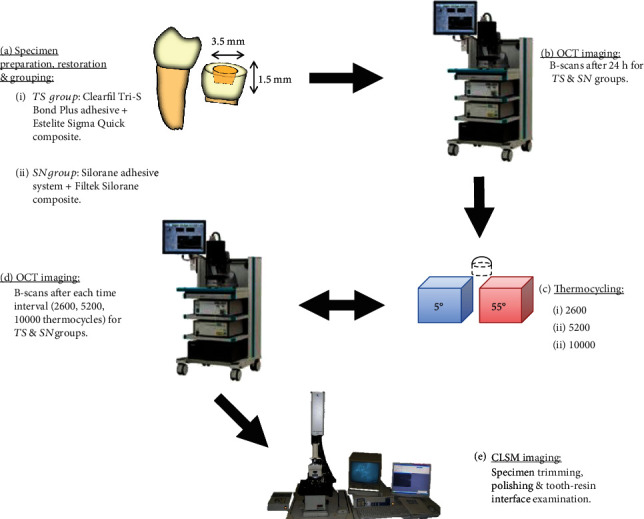
Schematic illustration showing the methodology of the study.

Afterward, all restored specimens were kept in distilled water for 24 h before SS-OCT baseline scanning. A schematic illustration of the research methodology is presented in Figure 1.

### 2.3. SS-OCT Imaging and Thermocycling

The technical specification of the SS-OCT imaging system (IVS2000, Santec, Japan) is described thoroughly elsewhere [23, 25]. Initially, each specimen was placed on a 3D micrometer stage. The occlusal surface was positioned parallel to the floor and perpendicular to the projected laser beam under the SS-OCT probe. Twelve sequential 2D scans (B-scans) were obtained at every 250 *μ*m for each specimen in the mesiodistal direction and considered baseline scans.

After that, all specimens were subjected to TC at 5°C and 55°C in a water bath for 2600, 5200, and 10000 cycles with a transfer time of 5 s and a dwell time of 30 s. Sequential B-scans were obtained for both groups at the end of each TC interval (2600, 5200, and 10000 cycles). Each B-scan corresponded to an image 8 mm × 6.6 mm dimensions (2001 pixels × 1019 pixels) obtained in approximately 100 ms.

### 2.4. OCT Image Analyses

Each B-scan was imported and managed by a plug-in macrofile coded in an image analysis software (ImageJ ver. 1.42q) in order to convert OCT data to a grayscale image according to the signal intensity value of each pixel in the matrix [22, 23, 26]. By analyzing the scans at the cavity floor, some areas showed high signal intensities that appeared as bright clusters of pixels, while others showed dark gray pixels. Based on previous reports, these bright clusters of pixels represent interfacial gaps [22, 23, 26]. OCT image analysis and gap quantification were carried out according to the protocol described by Bakhsh et al. [22, 26]. The imported OCT data was converted to an 8-bit grayscale image, subjected to a median filter (1px radius), and cropped to the area of interest, which included the whole cavity floor. Then, the image binarization function was employed to convert the cropped grayscale image to black and white image to determine the target pixels with significantly higher brightness compared to other dark pixels in the background. The transformed target pixels into black pixels on a white background at the cavity floor were considered as interfacial gaps, which were calculated in percentage according to the following equation:

(1) $$\begin{array}{c} \text{Gap}\left(\text{bright clusters length}\right)\%\frac{\text{total length of bright clusters at each slice }}{\text{length of the cavity floor at that slice}}\times 100. \end{array}$$

### 2.5. CLSM Imaging

After 10000 cycles, the imaged specimens were trimmed with SiC papers (600-200 grit) and polished with diamond polishing films and pasts in a descending size order (6.0–0.25 *μ*m) followed by interfacial examination using CLSM. Additional specimens for both groups were prepared, restored, OCT scanned, and trimmed after 24 h before being polished and examined under CLSM.

### 2.6. Statistical Analysis

Statistical analysis of the results was performed using a statistical software package (SPSS-2 for Windows: SPSS, USA). The values obtained from B-scans of each section were averaged and included in the statistical analysis. As the distribution of data was normal, a parametric test was performed. The difference in mean gap percentage between the tested groups at different TC intervals (baseline, 2600, 5200, and 10000 cycles) with a significance level defined as alpha = 0.05 was calculated.

## 3. Results

**Figure 2:**
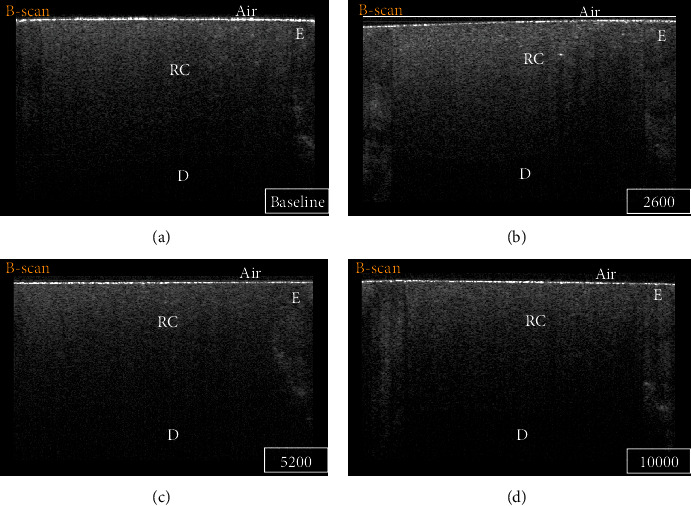
B-scan for a representative specimen of the TS group at different time intervals: (a) baseline, (b) 2600 TC, (c) 5200 TC, and (d) 10000 TC. Regardless of the time interval, the absence of bright bands of pixels with low backscattered reflection at the cavity floor indicates no loss of interfacial seal. E: enamel; D: dentin; RC: resin composite.

**Figure 3:**
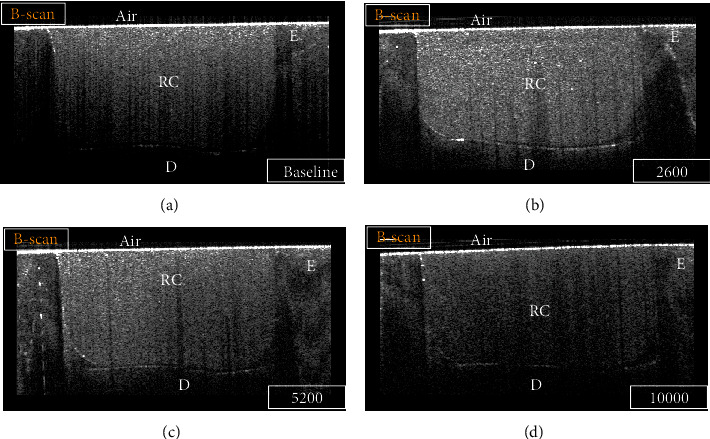
B-scan for a representative specimen of the SN group at different time intervals: (a) baseline, (b) 2600 TC, (c) 5200 TC, and (d) 10000 TC. Strong backscattered reflection at the cavity floor in some regions was demonstrated on the B-scan as bright bands of pixels that were considered an interfacial gap. In contrast, regions that did not show an increase in signal intensity at the tooth-resin interface indicated no loss of interfacial seal. E: enamel; D: dentin; RC: resin composite.

**Figure 4:**
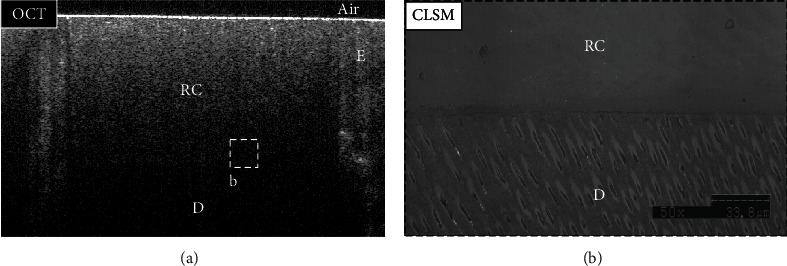
Representative images for the TS group. (a) B-scan showed low backscattered reflection at the cavity floor, including the target area (dotted box (b)). (b) A confirmatory CLSM image (×50 magnification) for the target area (dotted box (a)) showed no interfacial gap at the dentin-resin interface. E: enamel; D: dentin; RC: resin composite.

**Figure 5:**
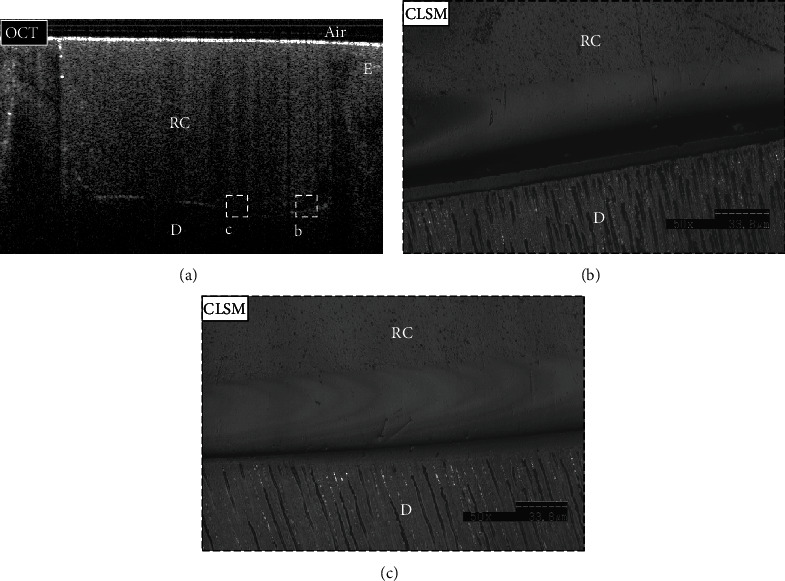
Representative images for the SN group (a–c). (a) B-scan showed low backscattered reflection at the cavity floor in some areas (dotted box (b)), while other areas showed intense backscattered reflection (dotted box (c)). (b, c) Confirmatory CLSM images (×50 magnification) corresponded to the obtained OCT findings. (b) The interfacial gap was detected within the adhesive layer and corresponded to the bright band of pixels in the OCT B-scan. (c) At the same time, it showed no interfacial gap in the dentin-resin interface of other areas, which was seen as dark pixels at the cavity floor in the presented OCT B-scan. E: enamel; D: dentin; RC: resin composite.

By analyzing SS-OCT images, some B-scans were showing high signal intensity (intense backscattered reflection) in the form of a bright cluster of pixels at the cavity floor at some regions (Figures 2 and 3). Other regions were not disclosing any abrupt changes in the pixel intensity values at the cavity floor, which corresponded to areas with no interfacial gaps between the bonded substrates under CLSM. Regions that showed high backscattered reflection under SS-OCT were confirmed as interfacial gap areas under CLSM (Figures 4 and 5).

A mixed ANOVA repeated measures with a Greenhouse-Geisser correction showed that the mean gap percentage differed significantly at different TC intervals (within subjects) for each adhesive system (*p* < 0.001). In addition, the type of adhesive system had a significant effect (between subjects) on the mean gap percentages within each TC interval (*p* < 0.001).

Student's independent *t-*test had shown statistically significant differences between tested groups, TS and SN, at each time interval (*p* < 0.001) (Table 2). Before subjecting the specimens to TC (baseline), the gap percentage in TS was smaller than in SN, with mean gap percentages of 2.22 (SD ± 1.78) and 20.55 (SD ± 14.11), respectively. After 2600 TC, the mean gap increased significantly in both groups, with a mean gap percentage of 3.03 (SD ± 2.24) in the TS group and 53.86 (SD ± 20.74) in the SN group. Then, the mean gap percentage of both groups decreased after 5200 and 10000 thermocycles, with mean gap percentages of 2.38 (SD ± 1.6) and 2.92 (SD ± 2.02) for the TS group and 31.6 (SD ± 17.21) and 36.10 (SD ± 16.64) for the SN group, respectively.

**Figure 6:**
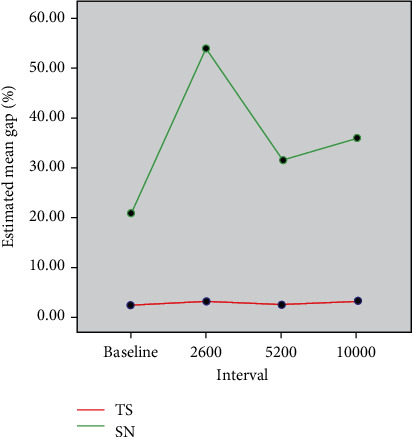
Comparison of estimated mean gap percentages at four time intervals between the TS and SN groups.

The estimated mean gap percentages at each time interval are given, as depicted in Figure 6.

## 4. Discussion

Thermocycling has been widely used in dental research as an aging technique. According to the International Organization for Standardization (ISO), the thermocycling regimen ISO/TS 11405 standard (2015) can be carried out while testing the quality of an adhesive bond between the restorative dental materials and the hard dental tissue. This includes the measurements from a tensile bond strength test, microleakage test, or marginal gap test. On the other hand, optical assessment of bonded composite restorations using OCT has been recently introduced to locate and quantify interfacial gaps in real time, which had altered the perception of polymerization shrinkage. Based on the current findings, TC is an appropriate method for understanding the durability of the bonded composite restorations. All bonding materials used in the present study with a TC method had clinically acceptable levels of marginal gaps.

In this study, SS-OCT was used to detect and monitor the changes in the gap length of bonded restorations subjected to thermal challenge. Gap detection by SS-OCT is based on analyzing the alteration of the backscattered signals that ascribed to the difference in the refractive indices (*n*) [22, 23]. Previous reports described the sudden increase in the signal intensity at the tooth-resin interface of bonded restorations as microgaps and a loss of interfacial seal [23]. Usually, bright bands of white clusters at the cavity floor are produced in B-scans when the OCT light passes through dissimilar substrates with a divergent index of refractions called “Fresnel” phenomena. On the contrary, this phenomenon is undetectable when OCT light passes through substrates with comparable refractive indexes. The index of refractions of enamel and dentin is relative to dental composites, ranging from 1.5~1.6 [23, 26–28]. Therefore, reduced signal interference will be detected on the signal intensity's profile (A-scan) in the absence of interfacial gaps. Regardless of the cause of the interfacial gaps, they are filled with either air or water, in a form of saliva or dentinal fluid, that have indexes of refractions equal to 1 and 1.3, respectively [22, 27]. This means that signal interference at the tooth-restoration interface is more intense when voids or microgaps are filled with air than with water. In this regard, the difference in signal intensities between the tested groups can be justified at different time intervals.

Dental adhesives are composed of hydrophilic and hydrophobic components that are designed to act as stress absorbers between hard dental tissues and dental composites. Several factors affect the restorations' durability in the oral cavity, which include the type of the substrate being bonded, surface area, rate and duration of the thermal challenge, thermal and hygroscopic expansion coefficients of the bonded substrates, magnitude and duration of the masticatory forces, and presence of defects within bonded substrates [29–31]. The coefficient of thermal and hygroscopic expansion of the bonded resins influenced the results of the current study. The results showed a divergence in gap measurements across the different time intervals. Before subjecting the specimens to TC, the mean gap percentage of TS and SN was equal to 2.22 and 20.55, respectively. After 2600 TC, the mean gap percentage had increased for both groups, which could be attributed to the generated interfacial stresses by thermal aging. Later, the gap in these bonded restorations had significantly decreased after 5200 and 10000 TC. Several studies showed that marginal integrity and bond strength of dental restorations decrease after short periods of TC and they ascribed their findings to the decrease in the resins' coefficient of thermal expansion [32–34]. However, other studies showed that prolonged thermal challenge would induce hygroscopic expansion in dental resin materials that improve the marginal integrity and compensate for the effect of polymerization shrinkage [35]. Both tested adhesives contain Bis-GMA and HEMA that may contribute to more water uptake and cause adhesive layer swelling, bond degradation under the influence of TC, which may explain the changes in the mean gap percentage in both groups over the different time intervals [36].

TP adhesive is a mild one-step self-etch adhesive (pH = 2.3) that contains phosphate monomer (10-MDP), which is characterized by a stable chemical bonding to hydroxyapatites [22]. This adhesive in the TS group was bonded to EQ composite with a volumetric filler loading equal to 71%. On the other hand, SA adhesive bonding system was bonded to FS composite in the SN group that was recognized as a low-shrinkage composite and contains siloxane core and four oxirane rings attached [37]. By examining the chemical composition of the two-step self-etch SA adhesive system, the adhesive primer with pH equals 2.7 contains a solvent, hydrophilic and hydrophobic monomers, fillers, initiators, and stabilizers. In contrast, the SA bond contains only hydrophobic monomers, fillers, and initiators. The SA adhesive layer was bonded to the FS composite that has a volumetric filler loading of around 55%. Although the FS composite includes a silorane ring responsible for reducing the shrinkage and optimizing its properties, the reduced filler loading has jeopardized composite adaptation. This low filler content had indirectly affected adhesive stability to resist the generated contraction stress against the cavity walls compared to the EQ composite. Moreover, the low pH of TP adhesive in the TS group would produce deep resin tags and a wide hybrid layer, unlike in the SN group. Thus, the difference in the monomer acidity and composite filler loading has influenced the study results and explains the difference in the mean gap percentage of both groups across the different time intervals.

It is noteworthy that although several studies demonstrated high bond strength with two-step self-etch adhesives in comparison to one-step self-etch adhesives, the primer utilized in the SN group resembles the components and action of one-step self-etch adhesives [38–40]. Two studies showed some voids and blister formation within the adhesive and between silorane bond and composite layers. A possible explanation would be related partly to incomplete solvent evaporation and uncured monomers that remain on the adhesive surface and partly to the high viscosity of this bonding system, which may trap air voids during the air-thinning step of the adhesive [38, 41]. These findings could explain the low performance of the SN group.

Within the limitations of the study, which include a narrow study design and a limited number of teeth—the null hypothesis was rejected.

## 5. Conclusion

An optimal composite adaptation can be achieved when the bonded restoration comprises a combination of an adhesive containing 10-MDP monomer and a considerable highly filled composite. Moreover, interfacial deformity under bonded composites can be monitored effectively using OCT.

## Figures and Tables

**Table 1:** List of the composition of the used materials in this study.

Material (Manufacturer) Code	Composition	Lot number	Fillers %	Manufacturer recommendation
Clearfil Tri-S Bond Plus adhesive One-step self-etch (Kuraray Noritake Dental, Japan) TP	*Adhesive*: MDP, Bis-GMA, HEMA, hydrophilic aliphatic dimethacrylate, hydrophobic aliphatic methacrylate, colloidal silica, sodium fluoride, dl-camphorquinone, accelerators, initiators, ethanol, water, others (pH = 2.3).	00007A	—	*Adhesive* (i) Apply bond for 10 s (ii) Dry with mild pressure air flow for 5 s (iii) Light cure for 10 s
Estelite Sigma Quick Universal composite (Tokuyama Dental, Japan) EQ	*Composite*: Bis-GMA, TEGDMA, silica–zirconia fillers, silica–titania fillers, photoinitiators.	J018	82% (wt.) 71% (vol)	*Composite* (i) Light cure the composite for 10 s, keeping the curing light tip within a distance of 2 mm from the surface
Silorane system adhesive Two-step self-etch (3M ESPE, USA) SA	*Primer*: phosphorylated methacrylates, Vitrebond copolymer, Bis-GMA, HEMA, water, ethanol, silane-treated silica filler, initiators, stabilizers (pH: 2.7).	N289224	—	*Primer* (i) Apply primer for 15 s (ii) Gentle air dry (iii) Light cure for 10 s
	*Bond*: hydrophobic dimethacrylate, phosphorylated methacrylates, TEGDMA, silane-treated silica filler, initiators, stabilizers.	N209848	—	*Bond* (i) Apply bond (ii) Gentle air dry (iii) Light cure for 10 s
Filtek Silorane Low shrinkage posterior composite (3M ESPE, USA) FS	*Composite*: silorane resin, CQ, iodonium salt, electron donor, quartz filler, yttrium fluoride, stabilizers, pigments.	N204592	76% (wt.) 55% (vol)	*Composite* (i) Light cure the composite for 20 s, keeping the curing light tip within a distance of 2 mm from the surface

HEMA: 2-hydroxyethyl methacrylate; Bis-GMA: bisphenol-A-diglycidyl methacrylate; TEGDMA: triethyleneglycoldimethacrylate; MDP: 10-methacryloyloxydecyl dihydrogen phosphate; CQ: camphorquinone; wt: weight; vol: volume.

**Table 2:** Mean gap percentage at each time interval using Student's independent *t-*test.

Time interval (thermocycles)	Group	Mean (%)	SD	Std. error	*p* value
Baseline	TS	2.22	1.78	0.30	<0.001
	SN	20.55	14.11	2.35	
2600	TS	3.03	2.24	0.37	<0.001
	SN	53.86	20.74	3.46	
5200	TS	2.38	1.60	0.27	<0.001
	SN	31.60	17.21	2.87	
10000	TS	2.92	2.02	0.34	<0.001
	SN	36.10	16.64	2.77	

## Data Availability

Data are available on request upon contacting the corresponding author by email (taabakhsh@kau.edu.sa).
